# High Capacity and Fast Kinetics of Potassium-Ion Batteries Boosted by Nitrogen-Doped Mesoporous Carbon Spheres

**DOI:** 10.1007/s40820-021-00706-3

**Published:** 2021-08-13

**Authors:** Jiefeng Zheng, Yuanji Wu, Yong Tong, Xi Liu, Yingjuan Sun, Hongyan Li, Li Niu

**Affiliations:** 1 Department of Materials Science and Engineering, College of Chemistry and Materials Science, Jinan University, Guangzhou, 510632 People’s Republic of China; 2 Center for Advanced Analytical Science, School of Chemistry and Chemical Engineering, Guangzhou University, Guangzhou, 510006 People’s Republic of China

**Keywords:** Potassium-ion batteries, Nitrogen doping, Mesoporous carbon anode, Kinetics

## Abstract

In view of rich potassium resources and their working potential, potassium-ion batteries (PIBs) are deemed as next generation rechargeable batteries. Owing to carbon materials with the preponderance of durability and economic price, they are widely employed in PIBs anode materials. Currently, porosity design and heteroatom doping as efficacious improvement strategies have been applied to the structural design of carbon materials to improve their electrochemical performances. Herein, nitrogen-doped mesoporous carbon spheres (MCS) are synthesized by a facile hard template method. The MCS demonstrate larger interlayer spacing in a short range, high specific surface area, abundant mesoporous structures and active sites, enhancing K-ion migration and diffusion. Furthermore, we screen out the pyrolysis temperature of 900 °C and the pore diameter of 7 nm as optimized conditions for MCS to improve performances. In detail, the optimized MCS-7-900 electrode achieves high rate capacity (107.9 mAh g^−1^ at 5000 mA g^−1^) and stably brings about 3600 cycles at 1000 mA g^−1^. According to electrochemical kinetic analysis, the capacitive-controlled effects play dominant roles in total storage mechanism. Additionally, the full-cell equipped MCS-7-900 as anode is successfully constructed to evaluate the practicality of MCS.

**Supplementary Information:**

The online version contains supplementary material available at 10.1007/s40820-021-00706-3.

## Highlights

- Nitrogen-doped mesoporous carbon spheres (MCS) are prepared as anode materials of potassium-ion batteries by a facile method.
- The MCS have larger interlayer spacing, high specific surface area, abundant mesoporous structures and nitrogen-doped active sites, achieving high-rate and long-cycle performances as anodes.
- The capacitive-controlled effects play dominant role in total storage mechanism and the MCS anodes are successfully applied to K-ion full-cells achieving high rate capacities.

**Supplementary Information:**

The online version contains supplementary material available at 10.1007/s40820-021-00706-3.

## Graphical abstract

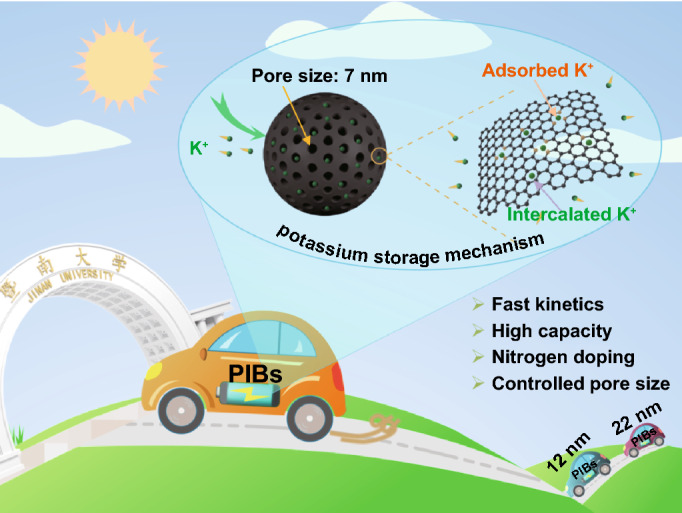

## Introduction

On account of high energy density and long-cycling lifespan, lithium-ion batteries (LIBs) have been proverbially utilized in numerous fields like increasing electric vehicles [1, 2]. With the rapid development of society, some issues such as uneven distribution of lithium (Li) and rising prices will inhibit their applications, especially in large-grid energy storage systems [3, 4]. Therefore, it is urgent to find new replaceable battery technologies. Based on inexpensive and resource-rich potassium salt, PIBs are more attractive [5]. It is worth noting that both PIBs and LIBs possess common foundation because of their similar working principle [6, 7]. Not only that, the potential versus SHE of K^+^/K (− 2.93 V) is nearly close to that of Li^+^/Li (− 3.04 V), enduing PIBs with conceivable high working voltage and energy density [8]. In addition, commercial graphite can be effectively used as PIBs anode, which lays the foundation for the further commercialization of PIBs [9]. Nonetheless, because the K-ion radius (1.38 Å) compared with that of Li ions (0.76 Å) is larger, apparent volume expansion and poor kinetics will appear in the course of potassiation, severely affecting the structure stability of electrode and the diffusivity of K ions [10]. Consequently, designing and preparing anode materials with high structural stability and fast kinetics are essential to deal with these thorny challenges.

It is reported that the present anode materials for PIBs mainly include carbon materials, metal-based chalcogenides (MCs), metal-based oxides (MOs) and alloy materials [5, 11–13]. Unfortunately, MCs, MOs and alloy materials are prone to generate larger volume variation than carbon materials during charge/discharge process, which is not conducive to maintaining their structural stability [14]. Comparatively speaking, durable and low-price carbon materials have obtained considerable attention. Carbon materials usually contain graphite, soft carbon, hard carbon, etc. Among them, graphite as anode electrodes having low theoretical capacity (only 279 mAh g^−1^) underwent inferior rate performance [15]. Some strategies have been taken to improve their electrochemical performances, such as expanded graphite [16]. Nevertheless, their electrochemical behaviors are still not so satisfying. In comparison, hard carbon with higher disorder degree and larger interlayer spacing is more conducive to improving electrochemical performances [17]. For instance, in 2016, hard carbon microspheres were first synthesized as anode materials for PIBs, achieving the capacity of 262 mAh g^−1^ at 28 mA g^−1^ [18]. Furthermore, Chen et al. [19] synthesized the porous hard carbon microspheres with sulfur and oxygen codoping as PIBs anodes which delivered the cycling capacities of 226.6 mA h g^−1^ at 50 mA g^−1^ over 100 cycles. For the purpose of obtaining better electrochemical performances, it is necessary to further modify hard carbon materials. It is a proved fact that porosity structure is beneficial to enhance the contact between electrolyte and anode material, providing more active sites, shortening K-ion diffusion distance and accommodating volumetric expansion [14, 20–23]. In addition, heteroatom doping, especially N doping, has been applied to increase active sites of carbon materials, so as to promote K-ion adsorption and increase their electronic conductivity [24–30]. Predictably, constructing carbon materials with porosity and N-doping positively impacts the performance improvement of the electrode materials.

Herein, the MCS were synthesized by facile and robust template method. According to the characterization, the as-prepared MCS possessed abundant mesopores, larger interlayer spacing in a short range, high specific surface area and plenty of N-doped active sites, which indicates their superiority of storing K. Based on experimental results, optimized conditions for ensuring high electrochemical performances of MCS were screened as the calcination temperature of 900 °C and the pore size of 7 nm. As a consequence, the optimized MCS-7-900 electrode exhibited high rate performance (107.9 mAh g^−1^ at 5000 mA g^−1^) and long-cycle stability (113.9 mAh g^−1^ after 3600 cycles at 1000 mA g^−1^). With respect to detailed K storage mechanism and K-ion diffusion kinetics, cyclic voltammetry (CV), electrochemical impedance spectroscopy (EIS) and galvanostatic intermittent titration (GITT) measurements were used to evaluate them. Given the kinetic analysis, the capacitive-controlled effects dominantly impacted K-ion storage. Deeply, theoretical calculation was carried out to understand the role of N doping. Encouragingly, full-cells were successfully constructed by taking advantage of MCS as anode materials, revealing their great potential for practical applications.

## Experimental Section

### Chemical Preparation

Colloidal silica was purchased from Sigma-Aldrich. Hydrochloric acid was obtained from Guangzhou Chemical Reagent Factory. Aniline (AR, 99.5%), ammonium persulfate (AR, 98.5%), sodium hydroxide (GR, 97%) and perylene-3, 4, 9, 10-tetracarboxylic dianhydride (PTCDA) (98%) were purchased from Shanghai Macklin Biochemical Co., Ltd. All chemicals were used as received. All aqueous solutions were prepared with ultrapure water (> 18.2 MΩ cm) from a Water Purifier System (UPR-II-10 T).

#### Preparations of MCS with Different Pore Sizes

The MCS were synthesized by a controlled self-assembly of hard template method in presence of polyaniline (PANI) using SiO_2_ nanoparticles as the sacrifice template. In brief, 8.525 mL SiO_2_ (30%, 7 nm) was dispersed in ultrapure water followed by addition of 10 mL 1 M HCl. After stirred for 10 min, 0.8 g aniline monomer was added into the above solution. Subsequently, 4 mL 1 M HCl solution with 2 g ammonium persulfate was added dropwise under vigorous stirring. The polymerization was performed in an ice bath for 24 h. After reaction, the product SiO_2_@PANI was collected by centrifugation and washed 3 times with ultrapure water. Afterward, the pyrolysis process of SiO_2_@PANI was conducted in a temperature programmable tube furnace at 900 °C under N_2_ flow. The SiO_2_ template was removed by the etching treatment in 2 M NaOH solution for 24 h. The resultant products are noted as MCS-7-900. Additionally, SiO_2_ nanoparticles with other sizes (12 or 22 nm), whose dosages were equal to that of SiO_2_ nanoparticles (7 nm) were used to prepare MCS-12-900 and MCS-22-900 by the above process, respectively.

#### Preparations of MCS at Different Pyrolysis Temperatures

To start with, the synthesis process of the product SiO_2_@PANI (7 nm) was the same as aforementioned steps. In the course of pyrolysis process, the difference was that two other temperatures (750 and 1050 °C) were carried out for the calcination of the product SiO_2_@PANI (7 nm). And the corresponding products are noted as MCS-7-750 and MCS-7-1050, respectively.

### Material Characterization

The morphology of MCS was characterized by the scanning electron microscopy (SEM) (Ultra-55) and transmission electron microscopy (TEM) (JEM-1400 flash). High-resolution transmission electron microscopy (HRTEM) was performed on a JEOL 2100F (Japan) with an acceleration voltage of 200 kV. The samples for TEM images were prepared by dropping the dilute colloidal suspension (~ 0.05 mg mL^−1^) onto a copper grid and dried in ambient air at room temperature. X-ray diffraction (XRD) patterns of MCS were recorded for 2*θ* with Cu K*α* radiation by X-ray diffractometer (miniflex 600). Raman spectroscopy was carried out on a LabRAM HR Evolution with a 532 nm laser excitation. The nitrogen adsorption–desorption measurements were performed on a Quantachrome ASiQwin-Autosorb IQ Station, and the bath temperature was 77.3 K (The outgas Temp. is 473.15 K). The specific surface area was calculated at 77.3 K using BET method. The element content was analyzed by X-ray photoelectron spectroscopy (Thermo Scientific *K*-Alpha +).

### Electrochemical Measurements

Half-cell: MCS were mixed with Ketjen Black and sodium carboxymethyl cellulose in a weight ratio of 80:10:10 and ground by agate mortar. Subsequently, the slurry was pasted on a copper foil and dried in vacuum. The MCS electrode with a diameter of 6 mm was obtained. Subsequently, the K metal was served as the counter electrode. The used electrolyte was 0.8 M KPF_6_ in EC/DEC = 1:1 vol%. The glass fiber (GF/A) from Whatman was used as the separator. Coin-type (CR2032) half-cells were assembled in an Ar-filled glove box with moisture and oxygen content of less than 0.2 ppm.

Full-cell: The PTCDA was mixed with Ketjen Black and sodium carboxymethyl cellulose at a ratio of 70:20:10 in weight on an aluminum foil, which was used as cathode with a diameter of 6 mm. The PTCDA cathode was prepotassiated before assembly. In addition, the MCS-7-900 anode was tested for 5 cycles to remove the irreversible capacity. The cathode material was 20% in excess in capacity than the anode material. The Whatman glass fiber (GF/D) was utilized as the separator. The electrolyte was 0.8 M KPF_6_ in EC/DEC = 1:1 vol%. The voltage window range for full-cell was 1.0–3.0 V. The specific capacity of the full-cell was calculated based on the mass of the active material in the anode. Moreover, the calculation of energy density of the full-cell was based on the mass of the active material of anode and cathode.

Electrochemical characterization: Electrochemical impedance measurements were tested in the frequency range from 100 kHz to 0.02 Hz by CHI760E Electrochemical Workstation. CV measurements were carried out at a scan rate of 0.1 mV s^−1^ and various scan rates by CHI660E Electrochemical Workstation. LAND-CT2001A battery-testing instruments were used to operate galvanostatic charge and discharge tests at room temperature under different current densities. All electrochemical measurements were performed in ambient conditions.

### Density Functional Theory (DFT) Calculations

All calculations for our study were conducted with the help of Vienna ab initio simulation package (VASP). Additionally, the electron interaction energy of exchange correlation was depicted by the Perdew–Burke–Ernzerhof generalized gradient approximation functional. The interaction in a long range can be better described by using Grimme’s semi-empirical DFT-D3 scheme in the computations. In detail, the plane wave cutoff was set to 450 eV. Furthermore, the Hellmann–Feynman forces convergence criterion on the atoms was set to be lower than 0.02 eV Å^−1^ during geometrical optimization. Tolerance of self-consistency was achieved at least 0.01 meV in the total energy. The Brillouin zone was sampled by taking advantage of Monkhorst–Pack method with Gamma centered to 3 × 3 × 1. In order to prevent interactions between the two repeated layers, a vacuum layer of 15 Å was built.

## Results and Discussion

### Structural Characterization and Analysis

**Fig. 1:**
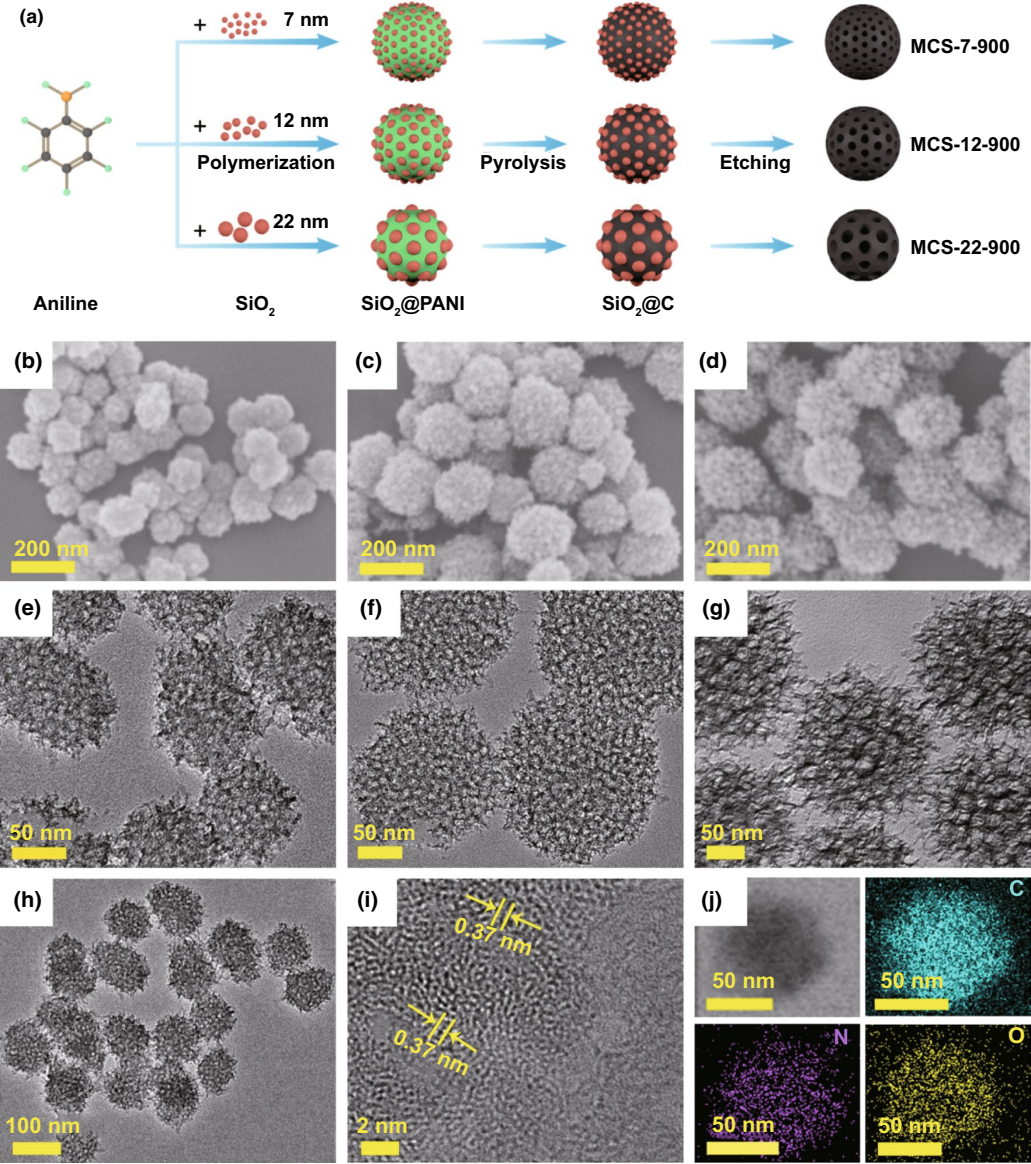
**a** Diagrammatic sketch of the preparation procedure of MCS-7-900, MCS-12-900 and MCS-22-900. SEM images of **b** MCS-7-900, **c** MCS-12-900 and **d** MCS-22-900. High-magnification TEM images of **e** MCS-7-900, **f** MCS-12-900 and **g** MCS-22-900. **h** TEM image of MCS-7-900. **i** HRTEM image of MCS-7-900. **j** Electron image with corresponding EDS mapping of MCS-7-900

Figure 1a clearly displays the synthesis process of MCS. Briefly, aniline and colloidal silica (7, 12, and 22 nm) were used to form SiO_2_@PANI composite by polymerization and self-assembly. And then, it was transformed into SiO_2_@C via pyrolysis at the temperature of 900 °C. Subsequently, the SiO_2_ was removed by using sodium hydroxide as etchant, and the correspondingly obtained MCS were marked as MCS-7-900, MCS-12-900, and MCS-22-900, respectively. Both TEM and SEM images clearly show the morphological and microstructural information of MCS. The as-prepared MCS possess relatively uniform size and spherical shapes in Fig. 1b–d. These features are further represented in Figs. 1h and S1, S2. Intuitively, abundant pores generating from the removal of SiO_2_ nanoparticles are obviously observed from Fig. 1e–g, which would provide shortened diffusion pathways for K ions and enhance the capacitive-controlled behaviors. In particular, the high-resolution TEM (HRTEM) image manifests that the MCS-7-900 not only have the evident characteristics of disordering, but also possess a large layer spacing of about 0.37 nm, which is beneficial to enhance K-ion storage behaviors (Fig. 1i). Besides, via conducting EDS mapping, it is clear to observe homogeneous distribution of C, N, and O elements in MCS-7-900 (Fig. 1j), demonstrating the successful formation of abundant N-doping sites. Besides, these oxygen elements appearing in the as-prepared samples were mainly due to the oxygen-doping effect of SiO_2_ hard template [31].

**Fig. 2:**
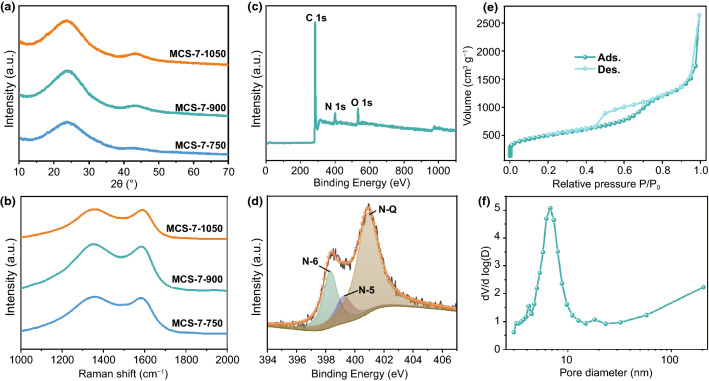
**a, b** XRD patterns and Raman spectra of MCS-7 calcined at 750, 900, and 1050 °C, respectively. **c, d** XPS survey spectrum and High-resolution N 1 s spectrum of MCS-7-900. **e, f** N_2_ adsorption–desorption isotherms as well as pore size distribution of MCS-7-900

XRD patterns, Raman spectra, X-ray photoelectron spectroscopy (XPS) as well as Nitrogen (N_2_) adsorption–desorption isotherms were exploited to verify the composition and structure of MCS. The XRD patterns exhibit two broad peaks located at around 24° and 43°, standing for the (002) and (101) planes, respectively (Fig. 2a). Accordingly, these wide peaks indicate the amorphous characteristics of MCS [32]. The Raman spectra (Fig. 2b) show two peaks near 1350 and 1583 cm^−1^, which presents D band (the disordered band) and G band (the graphitic band), respectively [33]. Based on both of bands, the intensity ratio (*I*_D_/*I*_G_) calculated by the absolute heights of the corresponding peaks can reveal the disorder degree of samples. The high *I*_D_/*I*_G_ values near 1.04 of MCS-7-750, MCS-7-900, and MCS-7-1050 implied that all of the as-prepared MCS possessed dominant disordered structure and partial graphitization.

Furthermore, the XPS survey spectrum of MCS-7-900 verifies the presence of C, N, and O elements (Fig. 2c), which is consistent with the results of the element mapping (Fig. 1g). Specifically, the atomic contents of C, N, and O elements in the MCS-7-900 were 87.39%, 5.27%, and 7.33%, respectively. Besides, XPS technology can reflect the chemical state of the elements. The high-resolution N 1 s spectrum of MCS-7-900 (Fig. 2d) displays three peaks situated at 398.3 eV (pyridinic N (N-6)), 399.3 eV (pyrrolic N (N-5)), and 401.0 eV (graphitic N (N–Q)) [34]. Among them, the relative percent of N-6, N-5, and N–Q are 23.69%, 13.48%, and 62.83%, which corresponds to the atomic content of 1.25%, 0.71%, and 3.31%, respectively (Fig. S3). In term of the high-resolution C 1 s spectrum of MCS-7-900 (Fig. S4a), the peaks are located at 284.8 eV (C–C), 286.2 eV (C=N), 288.1 eV (C–N), and 291.5 eV (COOH), which further demonstrates that N element has been doped into carbon layer [35, 36]. Additionally, the peaks from the high-resolution O 1 s spectrum of MCS-7-900 (Fig. S4b) are located at 531.1 eV (C=O), 532.3 eV (C–O), and 533.4 eV (COOH) [37]. The existence of O not only contributes to offering more active sites but also makes for enhancing the wettability [37]. Pore size distributions as well as specific surface area of the MCS-7-900, MCS-12-900, and MCS-22-900 were obtained from N_2_ adsorption–desorption isotherms. Figures 2e and S5a, b display the apparent hysteresis loops for the aforementioned samples, which implies the presence of mesopores. Furthermore, the specific surface area based on Brunauer–Emmett–Teller model of MCS-7-900 (1781 m^2^ g^−1^) was larger than that of MCS-12-900 (1261 m^2^ g^−1^) and MCS-22-900 (793 m^2^ g^−1^), helping to enhance the adsorption behaviors. The pore size distributions are in accordance with Barrett–Joyner–Halenda model and originate from the adsorption branch of isotherm, inferring the mesopores with around 7, 12, and 22 nm (Figs. 2f and S5c, d) [31]. Simultaneously, these pore sizes were highly consistent with the diameters of SiO_2_ templates we used.

### K-ion Half-Cell Performances

**Fig. 3:**
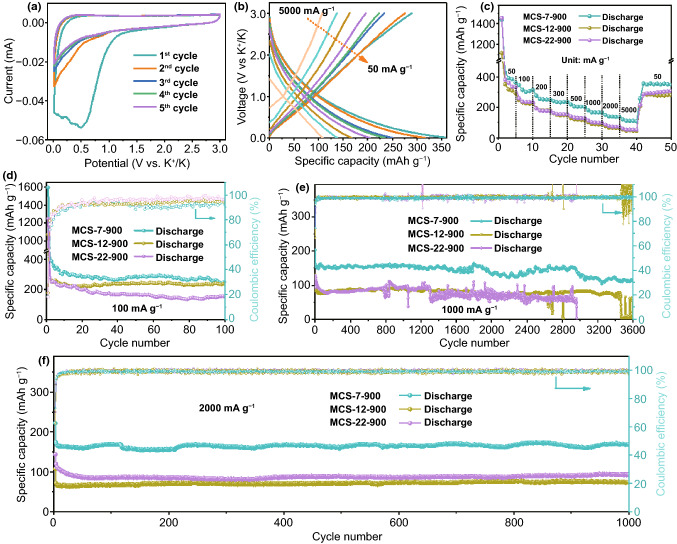
**a** CV profiles scanned at 0.1 mV s^−1^ and **b** the charge–discharge profiles in the range of 50–5000 mA g^−1^ of the MCS-7-900 electrode. Comparison of electrochemical performances of MCS-7-900, MCS-12-900 and MCS-22-900: **c** Rate performance; **d** Cycle performance at the current density of 100 mA g^−1^; Long-cycling performance at **e** 1000 mA g^−1^ and **f** 2000 mA g^−1^

Half-cells, whose counter electrodes were K metal, were assembled to assess the electrochemical performances of the MCS electrodes. For the sake of determining the appropriate calcination temperature, the relationship between the calcination temperature and the corresponding electrochemical properties has been explored. Compared with the electrochemical performances of MCS-7-750 and MCS-7-1050, the MCS-7-900 electrode releases the highest capacities, suggesting 900 °C is optimized temperature for this system (Figs. S6–S9). Given aforementioned contrastive results, the pyrolysis temperature of 900 °C stood out and was determined as optimized conditions for the performance improvement of MCS. More importantly, the different pore diameters likewise impinge on the electrochemical behaviors, so they are deserved to be focused. Figure 3a exhibits the initial five cycles of CV profiles for MCS-7-900 electrode at 0.1 mV s^−1^ with the voltage range of 0.01–3.0 V (versus K^+^/K). Among them, a salient peak located at 0.485 V in the cathodic scan of the first cycle resulted from the formation of solid electrolyte interphase (SEI) film [38]. Evidently, the shape of the subsequent cycles kept similar, and the fourth and fifth curves almost overlap, which implied the remarkable reversibility during charge/discharge process in the MCS-7-900 electrode. The CV profiles of other samples are similar to that of MCS-7-900 (Figs. S10 and S11). It is depicted that there are no evident voltage platforms in the charge–discharge curves of MCS-7-900 electrode at various current densities (Fig. 3b), accounting for the large specific surface area and predominant amorphous structure. Such behaviors were consistent with the characteristics of hard carbon [39].

In addition, the rate performances of MCS-7-900, MCS-12-900, and MCS-22-900 are compared in Fig. 3c. It was obvious that the MCS-7-900 electrode expressed the highest rate capacities. The discharge capacities of the MCS-7-900 electrode were 363.7, 315.9, 249.9, 235.2, 203.8, 166.5, 137.8, and 107.9 mAh g^−1^ at 50, 100, 200, 300, 500, 1000, 2000, and 5000 mA g^−1^, respectively. When returning to 50 mA g^−1^, the capacity still reached up to 351.4 mAh g^−1^, indicating the high reversible capacities of the MCS-7-900 electrode. Most importantly, after the pre-potassiation process, the first Coulomb efficiency of the MCS-7-900 electrode-based PIBs can be achieved at 78.81% (Fig. S12), which implies that the as-prepared materials have good potential in practical application. For further evaluating the K storage performances of MCS, galvanostatic current charge–discharge tests were carried out. Figure 3d exhibits the cycling performances of various electrodes at 100 mA g^−1^, where the MCS-7-900 electrode is superior to the other two samples. To be specific, the MCS-7-900 electrode remained the capacities of 254.4 mA g^−1^ after 100 cycles at 100 mA g^−1^. As a comparison, the capacities of the MCS-12-900 electrode and the MCS-22-900 electrode severally maintained 234.8 and 153.8 mAh g^−1^ after 100 cycles at the same current density. Moreover, long-term cycling tests are of the essence to evaluate cycling stability lifespan of electrodes. Note that all prepared electrodes first circulated about 2 cycles at 50 mA g^−1^ before testing at high current density. The comparison of long-cycle performance of the as-prepared samples at 1000 mA g^−1^ manifests the best cycling stability of MCS-7-900 (Fig. 3e). The capacity of the MCS-7-900 electrode decreased in the several cycles, which was attributed to the formation of SEI film [40]. Moreover, the capacities of the MCS-7-900 electrode went through up-and-down changes, which may be ascribed to the nonstationary temperature in an open environment.

Particularly, the cycling discharge capacity of the MCS-7-900 electrode was 113.9 mAh g^−1^ after 3600 cycles at 1000 mA g^−1^ with the average capacity decay of only 0.012% per cycle. As for MCS-12-900 and MCS-22-900, their performances are worse than that of MCS-7-900 and all samples appeared unstable phenomena during cycling process. Additionally, the MCS-7-900 electrode still hold highest capacity at higher current density compared with the MCS-12-900 and MCS-22-900 electrode (Fig. 3f). The capacity of the MCS-7-900 electrode was 169.6 mAh g^−1^ after 1000 cycles at 2000 mA g^−1^, while the capacities of MCS-12-900 and MCS-22-900 electrode were 73.9 and 92.8 mAh g^−1^ under the same condition, respectively. Moreover, the MCS-7-900 electrode realizes the capacity of 102.2 mAh g^−1^ after over 9000 cycles with the average capacity decay of 0.0035% per cycle at 5000 mA g^−1^ (Fig. S13). As mentioned above, the reasons for the excellent cycling life of the MCS-7-900 electrode are ascribed to smaller mesoporous size, large specific surface area, and N doping. Delightfully, a red light-emitting diode (LED) is driven by one half coin cell (Fig. S14), reflecting their practical application potential. As comparison, the electrochemical performances of the MCS anodes are superior to other reported anodes (Table S1).

### Storage Kinetics Analysis

**Fig. 4:**
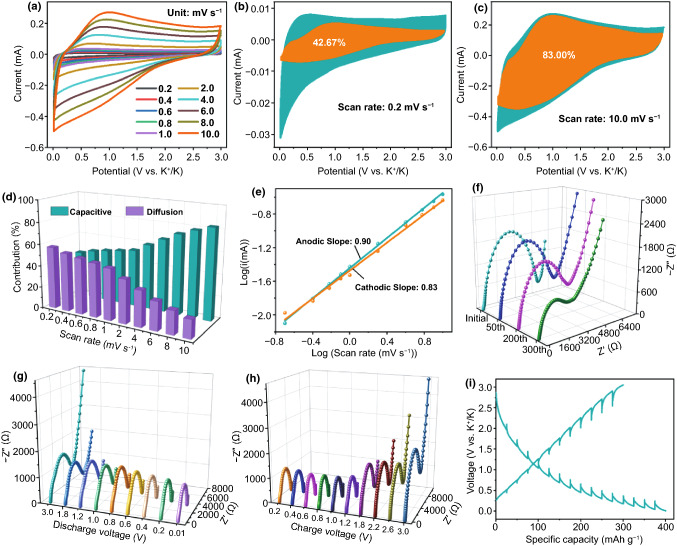
Properties of the MCS-7-900 electrode. **a** CV curves corresponding to various scan rates. Capacitive charge-storage contribution at the scan rates of **b** 0.2 mV s^−1^ and **c** 10 mV s^−1^. **d** Contribution ratios of capacitive- and diffusion-controlled effects. **e** Relationship between log (*i*) versus log (*v*) of the anodic and cathodic peaks. **f** Nyquist plots after different cycles from initial to 300th cycle at 1000 mA g^−1^. Ex-situ EIS plots of **g** discharge process and **h** charge process in the sixth cycle at 50 mA g^−1^. **i** GITT potential profiles for the MCS-7-900 electrode

CV technique was propitious to reflect the diffusion kinetics and reveal the storage mechanism. With the scan rates increasing from 0.2 to 10 mV s^−1^, the shapes of the CV curves occupy larger area, which illustrates the capacitive-controlled dominant characteristic (Figs. 4a and S15, S16) [17]. Clearly, a distortion from the basic shape appears at high scan rates (more than 2 mV s^−1^) in Fig. 4a, which may result from several aspects such as increased ohmic contribution and/or diffusion constraints [41]. According to these CV curves, the capacitive-controlled effects (*k*_1_*v*) and diffusion-controlled effects (*k*_2_*v*^0.5^) were confirmed by means of the following equation [42]:

1 $$i\left( V \right)/v^{0.5} = k_{1} v^{0.5} + k_{2}$$

where *i* stands for the current (mA) at a fixed potential, *k*_1_ and *k*_2_ present constants, and *ν* presents the scan rate (mV s^−1^). Subsequently, the plot of *i*(*V*)/*v*^0.5^ versus* v*^0.5^was fitting into a straight line so as to obtain *k*_1_ (slope) and *k*_2_ (intercept). As shown in Fig. S17, several fitting lines display the high goodness of fit at the selected voltages. In order to visualize the detailed contributions intuitive, the azure blue and orange regions correspond to diffusion- and capacitive-controlled contribution, respectively. It is apparent that capacitive-controlled contributions grow from 42.67 to 83.00% as the scan rate increased from 0.2 to 10.0 mV s^−1^ (Figs. 4b, c and S18). Figure 4d exhibits the evident trend of increasing capacitive-controlled contributions and decreasing diffusion-controlled contributions. It demonstrates that the capacitive-controlled effects gradually dominate in the storage mechanism with the increase of scan rates, which is advantageous to achieve high rate performance. Additionally, the power-law relationship between the scan rate as well as the peak current conforms to the following formula [43]:

2 $$i = av^{b}$$

where *i* presents the peak current (mA), and *v* is the scan rate (mV s^−1^). Based on the linear plot of log (*i*) versus log (*v*), its slope is the value of b. The calculated results show that the anodic slope and cathodic slope are 0.90 and 0.83, respectively (Fig. 4e), suggesting the capacitive-controlled effects play dominant roles in K storage. These aforementioned phenomena mainly resulted from high specific surface area, ample mesoporous structures and N doping.

Furthermore, EIS was employed to analyze the charge-transfer resistance and ion diffusion of the MCS-7-900 electrode. Figure 4f displays that all spectra include semicircle in the high frequency to medium-frequency and the Warburg line in the low-frequency according to Nyquist plots, both of which present the charge-transfer resistance (*R*_ct_) and the ion diffusion, respectively [44]. With the cycle numbers increasing, the values of *R*_ct_ based on equivalent circuit model (Fig. S19) gradually decreases, successively presented as 4600 (initial), 4086 (50th), 630.1 (200th), and 550 (300th) Ω. Hence, this tendency may be ascribed to the impact of self-activating [45]. Subsequently, ex-situ EIS was used to further reflect the electrochemical kinetics of the MCS-7-900 electrode by obtaining the Nyquist plots corresponding to a series of discharge/charge voltages in the sixth cycle (Fig. 4g, h). Based on the fitting results of ex-situ EIS, the values of *R*_ct_ at the different voltages were exhibited (Fig. S20), where the *R*_ct_ gradually decreases during the discharge process and increases within the charge process. The decreasing *R*_ct_ during discharge process may be due to the formation of stable SEI film and potassiation. After that, the depotassiation may lead to the increasing *R*_ct_ in the course of charge process [46, 47].

Besides, GITT means makes for obtaining the diffusion coefficient (*D*) and investigating K-ion diffusion kinetics of the MCS-7-900 electrode during charge/discharge process (Fig. 4i) based on Eqs. 3 and 4 [17].

3 $$D = \frac{4}{\pi \tau }\left( {\frac{{m_{{\text{B}}} V_{{\text{M}}} }}{{M_{{\text{B}}} S}}} \right)^{2} \left( {\frac{{\Delta E_{{\text{S}}} }}{{\Delta E_{{\uptau }} }}} \right)^{2}$$

4 $$\rho = \frac{1}{{V_{{{\text{total}}}} + \frac{1}{{\rho_{{{\text{carbon}}}} }}}}$$

where *τ* is the duration of the current pulse; *m*_B_ presents the mass loading of electrode active material; *S* stands for the geometric area of the electrode. As illustrated in Fig. S21, *ΔE*_S_ presents the quasi-thermodynamic equilibrium potential difference between before and after the current pulse, and *ΔE*_*τ*_ presents the potential difference in the course of the current pulse; *V*_M_ and *M*_B_ are the molar volume and the molar mass of the materials, respectively. *ρ* (g cm^−3^) is the density of the materials; *V*_total_ (cm^3^ g^−1^) is the total pore volume originating from the N_2_ adsorption–desorption isotherms; *ρ*_carbon_ presents the true density of carbon (2 g cm^−3^). Note that the value of *M*_B_/*V*_M_ is gained from the density of the materials according to Eq. 4. During discharging process, the diffusion coefficient (*D*) decreases slowly and reaches up to lowest value, then rises a little and subsequently declines (Fig. S22a).

These behaviors were able to be explained by the following reasons. First of all, from the perspective of surface and defects, both of them first came into contact with K ions. And these received ions would generate resistance for the transportation of subsequent ions, resulting in a continuous descending trend of the diffusion coefficient. Secondly, the internal pore adsorption would increase the diffusion coefficient [48, 49]. Thirdly, with the diffusion distance lengthening, the coulomb repulsion of the existing ions gradually strengthened, causing the curve drops [33]. It is worth noting that the charge curve is very similar to the discharge profile, which implies high reversibility of the MCS-7-900 electrode (Fig. S22b). Additionally, it is convenient to comprehend the structural changes by ex-situ XRD measurement. As for the position of (002) peak, it can shift toward lower angle when discharging to 0.01 V and shift back to near the pristine location after charging to 3.0 V (Fig. S23) [50]. This change process indicated the outstanding reversibility of the MCS-7-900 electrode, which was conducive to achieving long-cycle lifespan. Besides, some evident peaks aligned with the position of KHCO_3_ (PDF No. 70-1168), which may be derived from the irreversible side reaction; especially, the peak near to 23° may correspond to the residual electrolyte KPF_6_ [51].

### DFT Calculations

**Fig. 5:**
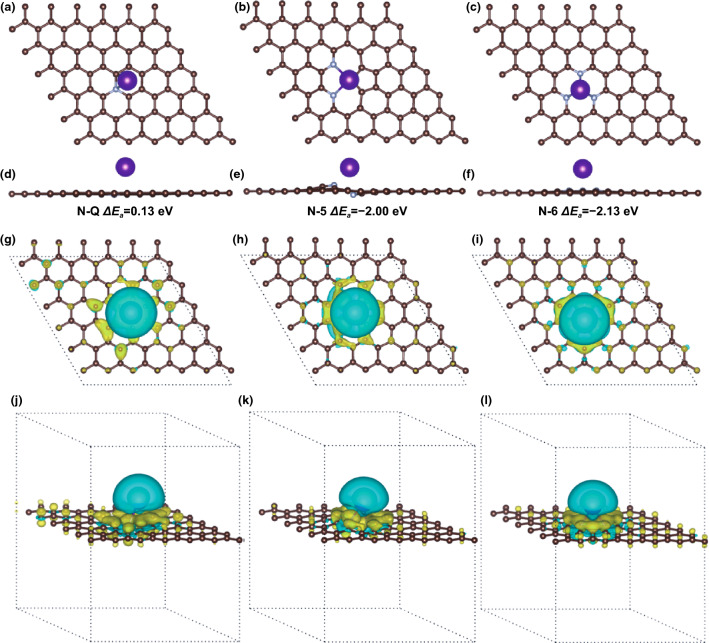
K-adsorption theoretical simulations based on various N-doped structures. Top and side view of a K atom adsorbed in the N-Q (**a, d**), N-5 (**b, e**) and N-6 (**c, f**) structures and the corresponding adsorption energy. Top and side view of K-adsorption Electron density differences for the N-Q (**g, j**), N-5 (**h, k**) and N-6 (**i, l**) structures. Not that, C, N and K atoms are presented by brown, silver and purple balls, respectively. Increased and decreased electron densities are presented by yellow and blue regions, respectively

Based on DFT, the theoretical simulations using first-principles calculations were employed to reflect the effects of the N-doped structures adsorbing K and explain relevant experimental phenomenon. Figure 5a, b shows the top view and side view of N–Q structure with the adsorption energy (*ΔE*_*a*_) of 0.13 eV. The positive *ΔE*_*a*_ of N-Q structure may be ascribed to the electron-richness of N–Q, negatively affecting K-adsorption [33]. Besides, the N-5 structure coexisting with another two N atoms possesses the *ΔE*_*a*_ of − 2.00 eV, which can enhance K-adsorption tendency (Fig. 5b, e). Furthermore, N-6 structure with three N atoms has the *ΔE*_*a*_ of − 2.13 eV, indicating this structure is more conducive to K-adsorption (Fig. 5c, f). As shown in Fig. S24a, b, the pristine carbon structure absorbing one K atom have the *ΔE*_*a*_ of − 0.25 eV, which signifies this structure is advantageous to promote K-adsorption. Deeply, electron density difference helps to comprehend the bonding nature of the adsorbed K atoms (Figs. 5g–l and S24c, d). Base on both K atoms and the carbon layers’ intermediate region, all structures including N-doped and the pristine carbon exhibited a net gain of charge, meaning that a charge transfer from the adsorbed K to its nearest neighboring C atoms [52]. Furthermore, it is noteworthy that N-doping sites of aforementioned structures display higher charge density. Among them, the effects of N-5 and N-6 were more evident [36]. As for the pristine carbon, the bonding carbons were inclined to accumulate charge density.

### K-ion Full-Cell Performances

**Fig. 6:**
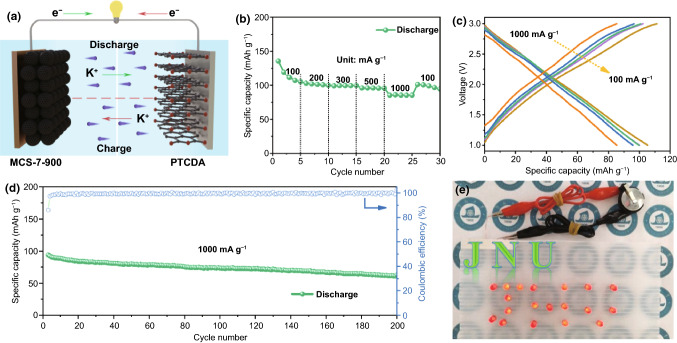
**a** Diagrammatic sketch of MCS-7-900//PTCDA K-ion full-cell. **b** Rate performance of full-cell. **c** Charge–discharge profiles of full-cell at 100–1000 mA g^−1^. **d** Long-term cycling performance of full-cell at 1000 mA g^−1^. **e** Red LEDs with the working voltage range of 1.8–2.0 V displaying “JNU” driven by one full-cell

K-ion full-cells are assembled by exploiting the MCS-7-900 as anode and the 3, 4, 9, 10-perylenetetracarboxylic dianhydride (PTCDA) as cathode to verify their application value, and schematic diagram is shown in Fig. 6a. The reversible capacity of PTCDA cathode is around 120 mA g^−1^ after 5 cycles at 50 mA g^−1^ (Fig. S25). Symmetric CV curve of MCS-7-900//PTCDA full-cell at 0.1 mV s^−1^ in the voltage window of 1.0–3.0 V demonstrates the remarkable reversibility of electrochemical reaction (Fig. S26a). To some extent, with the scan rates increased, the symmetry of CV profiles will be affected (Fig. S26b). Rate performance of MCS-7-900//PTCDA full-cell is exhibited in Fig. 6b, c. The rate capacities were 105.3, 100, 99.6, 95.8, and 85.4 mAh g^−1^ (based on the active material of anode) at 100, 200, 300, 500, and 1000 mA g^−1^, respectively. When returned back to 100 mA g^−1^, the capacity smoothly maintained 94.5 mAh g^−1^, suggesting high reversibility of MCS-7-900//PTCDA full-cells.

Additionally, the average capacity decay of MCS-7-900//PTCDA full-cell is 0.168% per cycle after 200 cycles at 1000 mA g^−1^ (Fig. 6d). Compared with that of half-cells with the MCS-7-900 anode, higher capacity decay of full-cells may result from the mismatched capacity ratio between anode and cathode [53]. Nonetheless, the electrochemical performances of MCS-7-900//PTCDA full-cell are comparatively outstanding among some reported full-cells (Table S2). The energy density of MCS-7-900//PTCDA full-cell was 59.7 wh kg^−1^, which was higher than that of reported potassium-ion storage devices [54–59]. Intuitively, one full-cell could light up a series of red LEDs presenting “JNU” (Fig. 6e), achieving the real application of full-cell.

## Conclusions

In this work, the MCS were synthesized via a simple and robust template method, demonstrating larger interlayer spacing in a short range, high specific surface area, abundant mesoporous structures and N-doped active sites. Accordingly, the high specific surface area and plentiful mesopores are beneficial to enhance the contact between electrolyte and electrode materials, promoting the K-ion transfer and improving the adsorption behaviors. N doping contributes to increasing plentiful active sites. For the purpose of obtaining optimal performance, pore size and pyrolysis temperature are all explored. It is concluded that the MCS with the pore diameter of 7 nm, derived from the temperature of 900 °C exhibit the optimized electrochemical performances. Accordingly, the optimized MCS-7-900 electrode achieves high rate performance (107.9 mAh g^−1^ at 5000 mA g^−1^) as well as superior cycle life (113.9 mAh g^−1^ after 3600 cycles at 1000 mA g^−1^ with the average capacity decay of 0.012%). According to the CV test, the capacitive-controlled effects are dominant in the storage mechanism of the MCS. In addition, the MCS have a high diffusion coefficient, indicating their low diffusion resistance. The simulating results reveal that the MCS with adsorbability are mainly related to pyridine and pyrrole N doping, which is instrumental in improving the K-ion adsorption. Inspiringly, the MCS electrode is successfully applied to K-ion full-cell. Our work can provide ideas for improving the electrochemical performances of PIBs.

## Supplementary Information

Below is the link to the electronic supplementary material.

Supplementary file1 (PDF 2257 KB)
