# Supplementary material for: High Capacity and Fast Kinetics of Potassium-Ion Batteries Boosted by Nitrogen-Doped Mesoporous Carbon Spheres
Source: Nanomicro Lett. 2021 Aug 13;13:174. doi: 10.1007/s40820-021-00706-3 (PMC8363726; doi:10.1007/s40820-021-00706-3)
Supplement: Supplementary file 1 — Supplementary file1 (PDF 2257 KB) [file 40820_2021_706_MOESM1_ESM.pdf]

Supporting Information for

High Capacity and Fast Kinetics of Potassium-Ion Batteries Boosted by Nitrogen-Doped Mesoporous Carbon Spheres

Jiefeng Zheng[1], Yuanji Wu[1], Yong Tong[1], Xi Liu[1], Yingjuan Sun[1,*], Hongyan Li[1,*] and Li Niu[2]

[1]Department of Materials Science and Engineering, College of Chemistry and Materials Science, Jinan University, Guangzhou 510632, P. R. China

[2]Center for Advanced Analytical Science, School of Chemistry and Chemical Engineering, Guangzhou University, Guangzhou 510006, P. R. China

*Corresponding authors. E-mail: lihongyan@jnu.edu.cn (Hongyan Li), yjsun@jnu.edu.cn (Yingjuan Sun)

Supplementary Tables and Figures

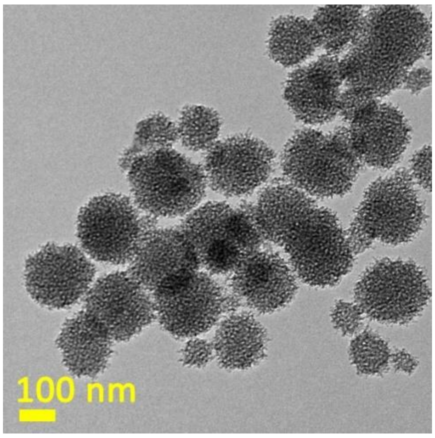

Fig. S1 TEM image of MCS-12-900

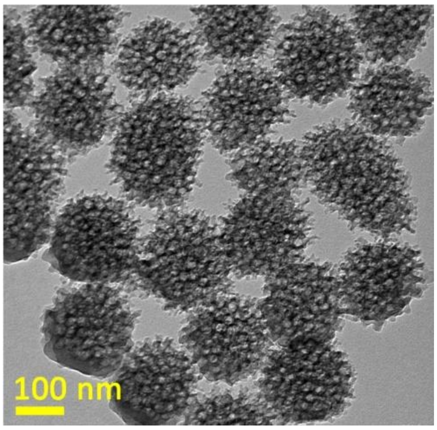

Fig. S2 TEM image of MCS-22-900

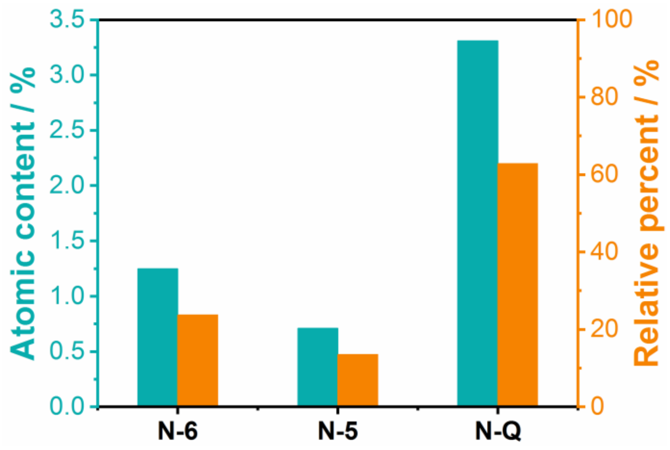

Fig. S3 Atomic content and relative percent of nitrogen from MCS-7-900

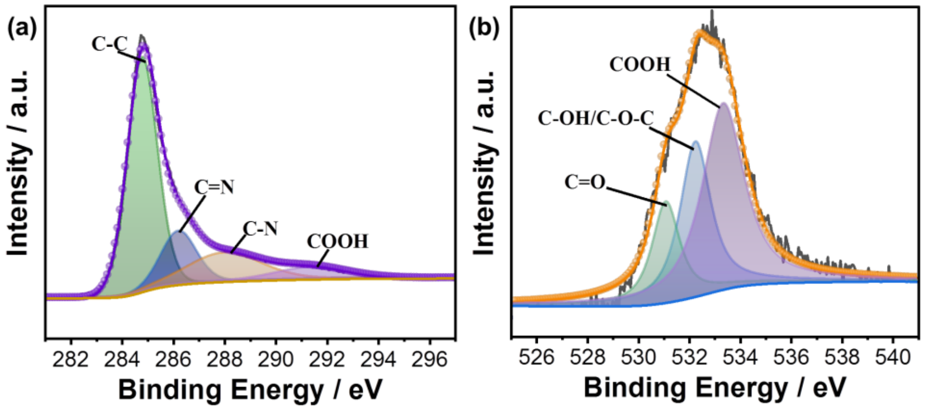

Fig. S4 a High-resolution C 1s spectrum of MCS-7-900. **b** High-resolution O 1s spectrum of MCS-7-900

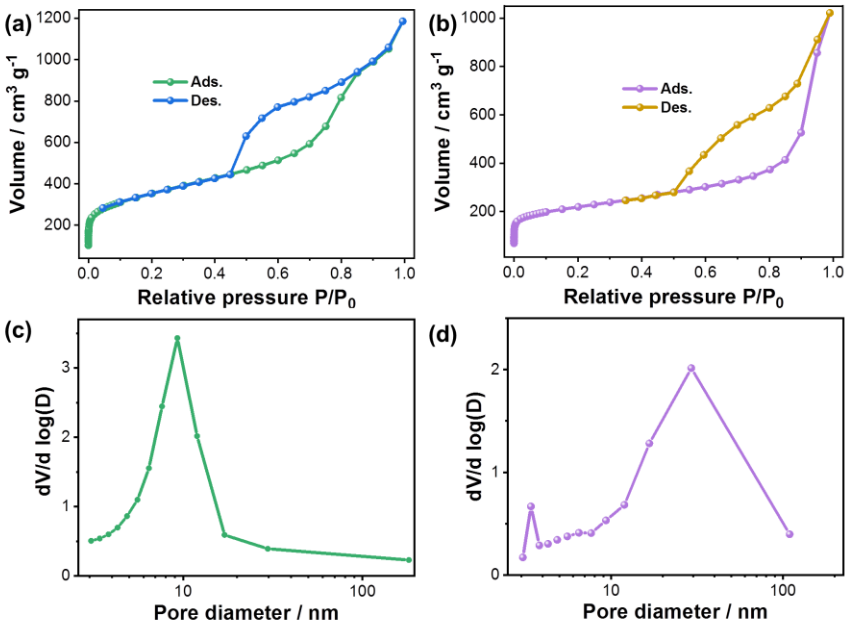

Fig. S5 a, **c** N_2 adsorption-desorption isotherms and pore size distribution of MCS-12-900. **b**, **d** N_2 adsorption-desorption isotherms and pore size distribution of MCS-22-900

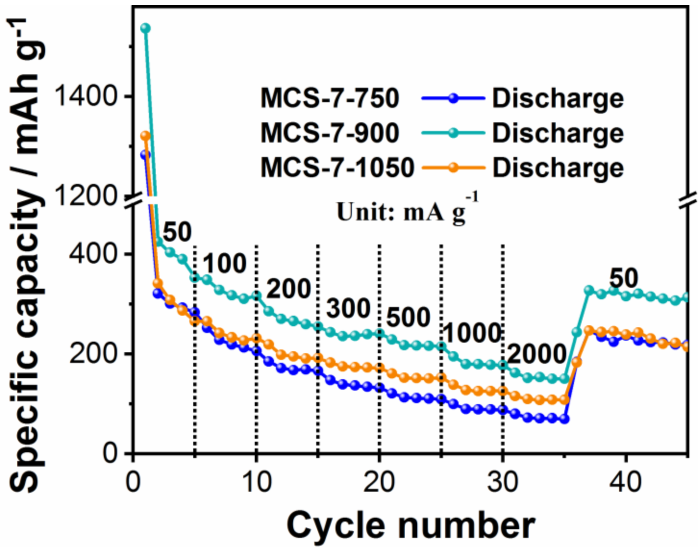

Fig. S6 Comparison of rate performances of MCS-7-750, MCS-7-900 and MCS-7-1050 at various current densities

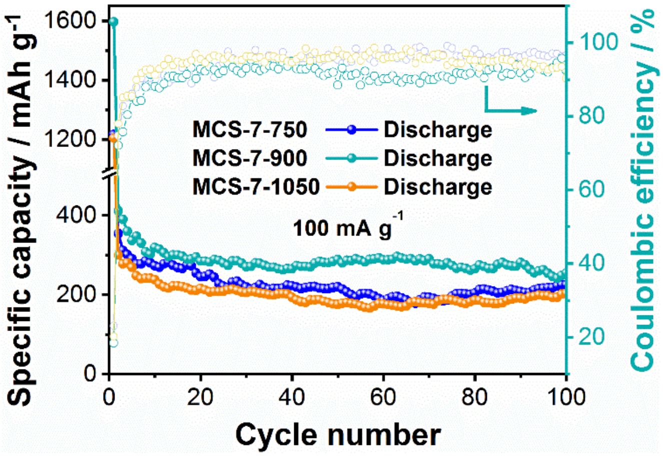

Fig. S7 Comparison of cycling performances of MCS-7-750, MCS-7-900 and MCS-7-1050 at 100 mA g^{-1}

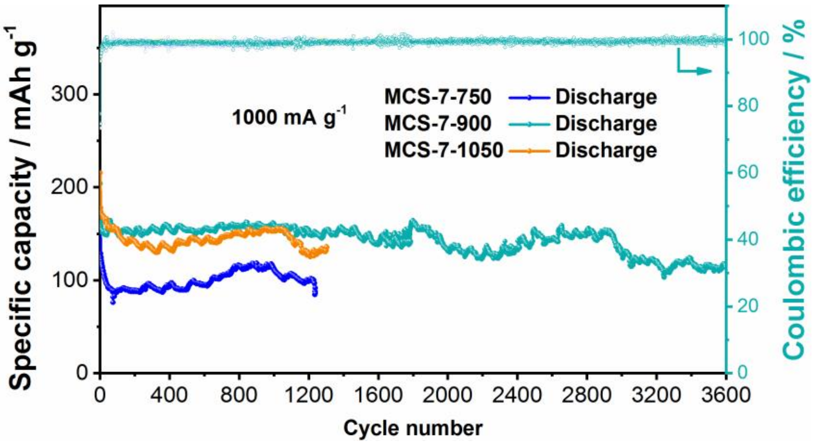

Fig. S8 Comparison of Long cycling performances of MCS-7-750, MCS-7-900 and MCS-7-1050 at 1000 mA g^{-1}

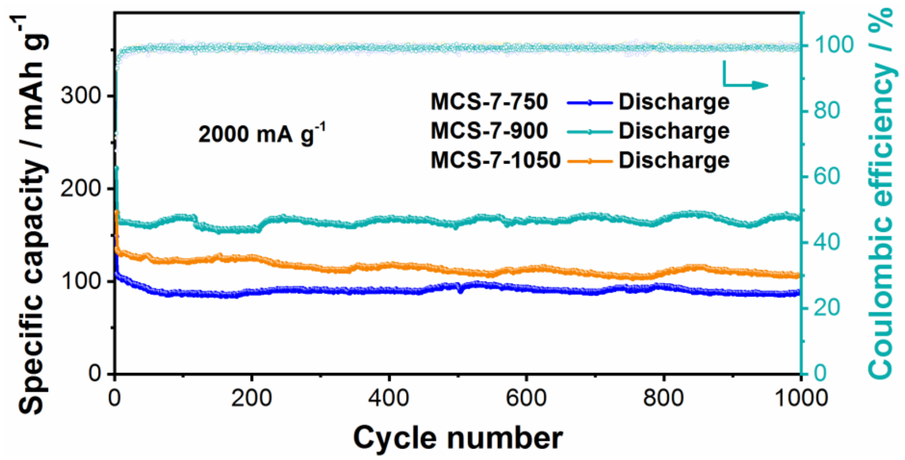

Fig. S9 Comparison of Long cycling performances of MCS-7-750, MCS-7-900 and MCS-7-1050 at 2000 mA g^{-1}

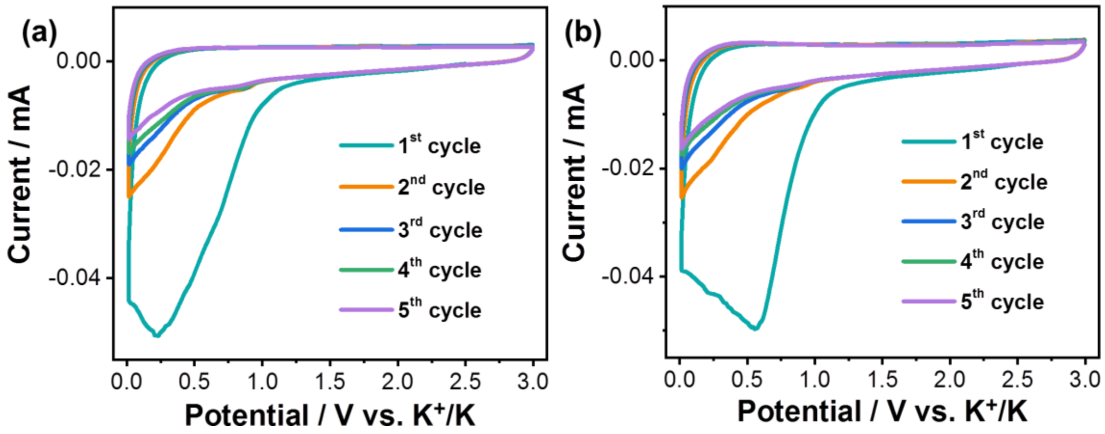

Fig. S10 CV curves of **a** MCS-7-750 and **b** MCS-7-1050 at 0.1 mV s^{-1}

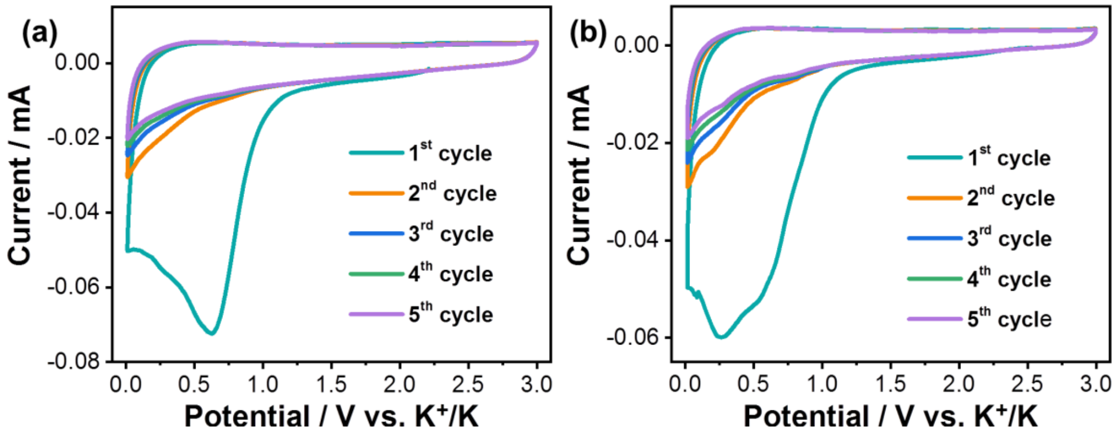

Fig. S11 CV curves of **a** MCS-12-900 and **b** MCS-22-900 at 0.1 mV s^{-1}

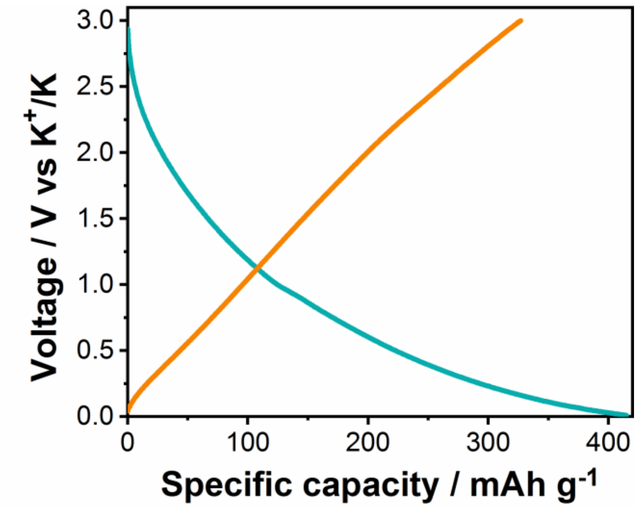

Fig. S12 Charge-discharge profiles of the MCS-7-900 electrode after pre-potassiation at the current density of 50 mA g^{-1}

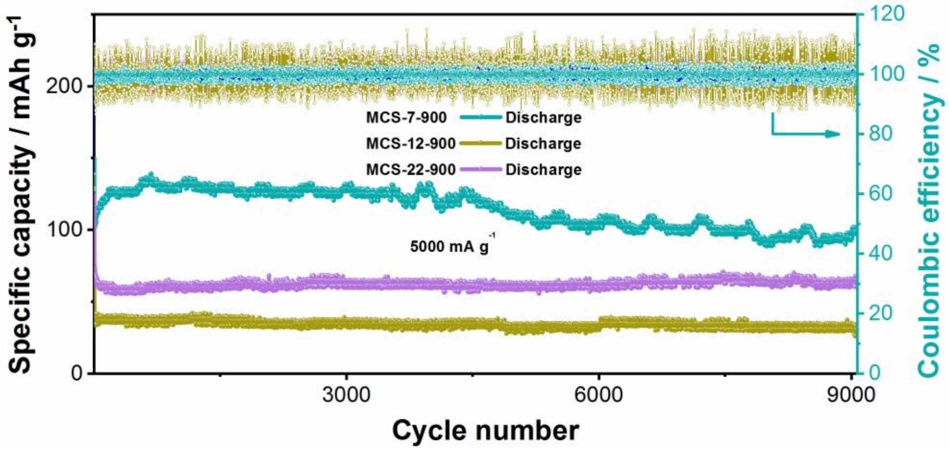

Fig. S13 Long cycling performance of MCS-7-900, MCS-12-900 and MCS-22-900 at 5000 mA g^{-1}

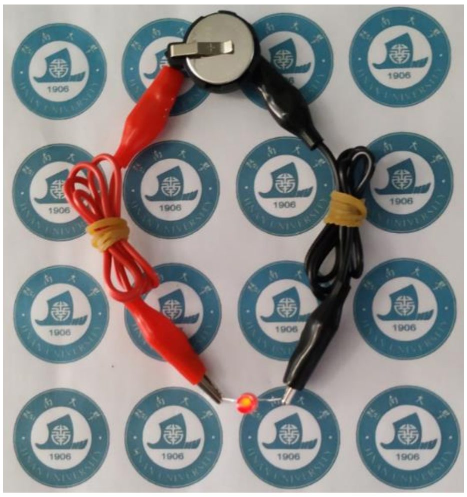

Fig. S14 A red LED light with the working voltage range of 1.8 V-2.0 V driven by one half cell with MCS-7-900 electrode and K metal as counter electrode

Table S1 Comparison of MCS-7-900 and other developed anodes about rate performance and cycling performance

Materials	Rate performance	Cycling performance	Refs.
MCS-7-900	137.8 mAh g^{-1} at 2000 mA g^{-1}	169.6 mAh g^{-1} after 1000 cycles at 2000 mA g^{-1}	This work
CoP@CNFC	30.8 mAh g^{-1} at 2000 mA g^{-1}	56.7 mAh g^{-1} after 2500 cycles at 180 mA g^{-1}	[S1]
PI-700-P28	133 mAh g^{-1} at 2000 mA g^{-1}	119.5 mAh g^{-1} after 500 cycles at 500 mA g^{-1}	[S2]
NSC	102.5 mAh g^{-1} at 2000 mA g^{-1}	105.2 mAh g^{-1} after 600 cycles at 2000 mA g^{-1}	[S3]
RHC-1100	62.72 mAh g^{-1} at 1000 mA g^{-1}	103.77 mAh g^{-1} after 500 cycles at 500 mA g^{-1}	[S4]
Co-NC	80.2 mAh g^{-1} at 2000 mA g^{-1}	78.5 mAh g^{-1} after 1000 cycles at 1000 mA g^{-1}	[S5]
SC-700	124.7 mAh g^{-1} at 2000 mA g^{-1}	≈85 mAh g^{-1} after 300 cycles at 500 mA g^{-1}	[S6]
N-PC	135 mAh g^{-1} at 1000 mA g^{-1}	≈111 mAh g^{-1} after 100 cycles at 1000 mA g^{-1}	[S7]
MEG-3	88 mAh g^{-1} at 1500 mA g^{-1}	152 mAh g^{-1} after 200 cycles at 100 mA g^{-1}	[S8]
N, S-C/SnS_2 nanosheet	104.5 mAh g^{-1} at 2000 mA g^{-1}	105.8 mAh g^{-1} after 200 cycles at 2000 mA g^{-1}	[S9]
SC-2	65.8 mAh g^{-1} at 1000 mA g^{-1}	80.8 mAh g^{-1} after 2000 cycles at 500 mA g^{-1}	[S10]

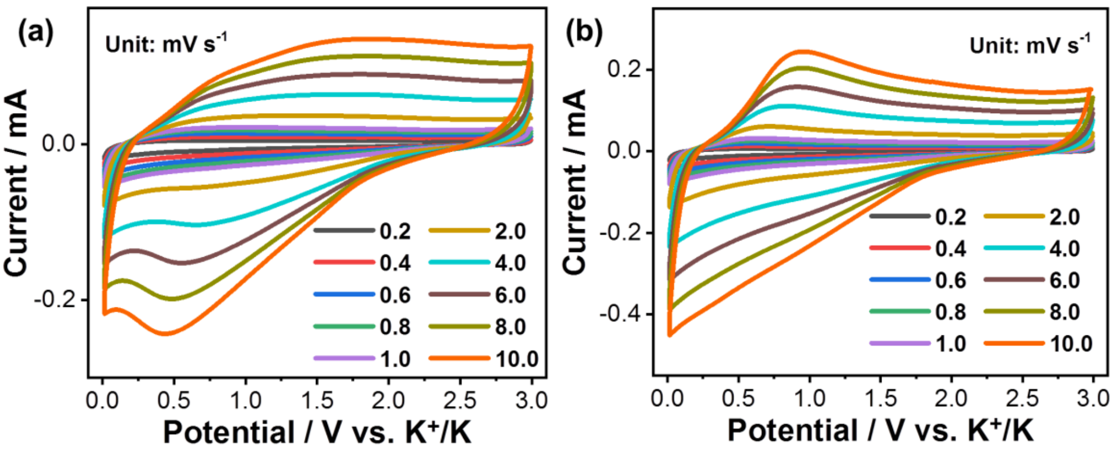

Fig. S15 CV curves at various scan rates of **a** MCS-7-750 and **b** MCS-7-1050

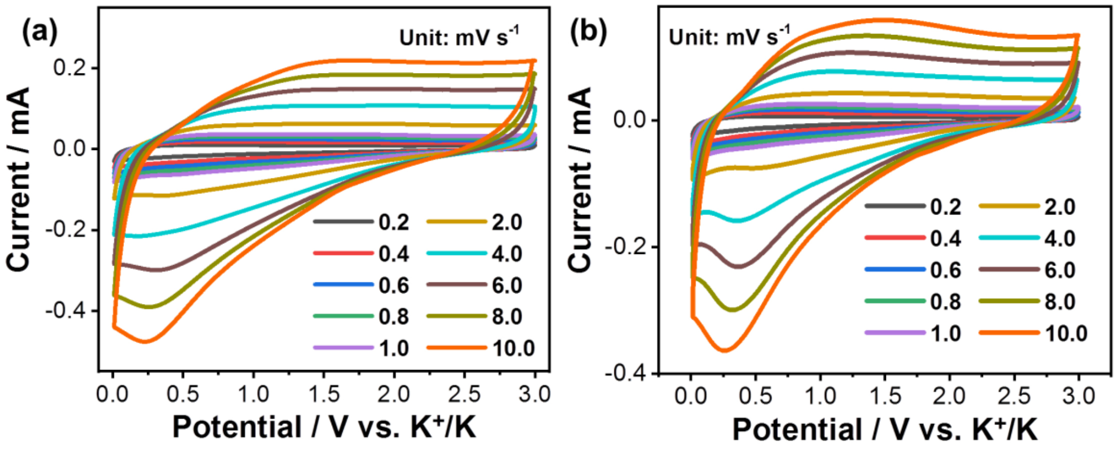

Fig. S16 CV curves at various scan rates of **a** MCS-12-900 and **b** MCS-22-900

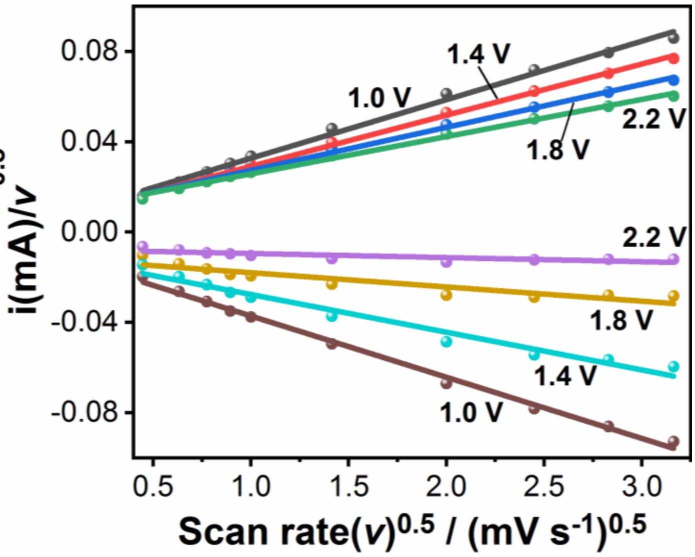

Fig. S17 Relationship between i(V)/$v^{0.5}$ *vs.* $v^{0.5}$

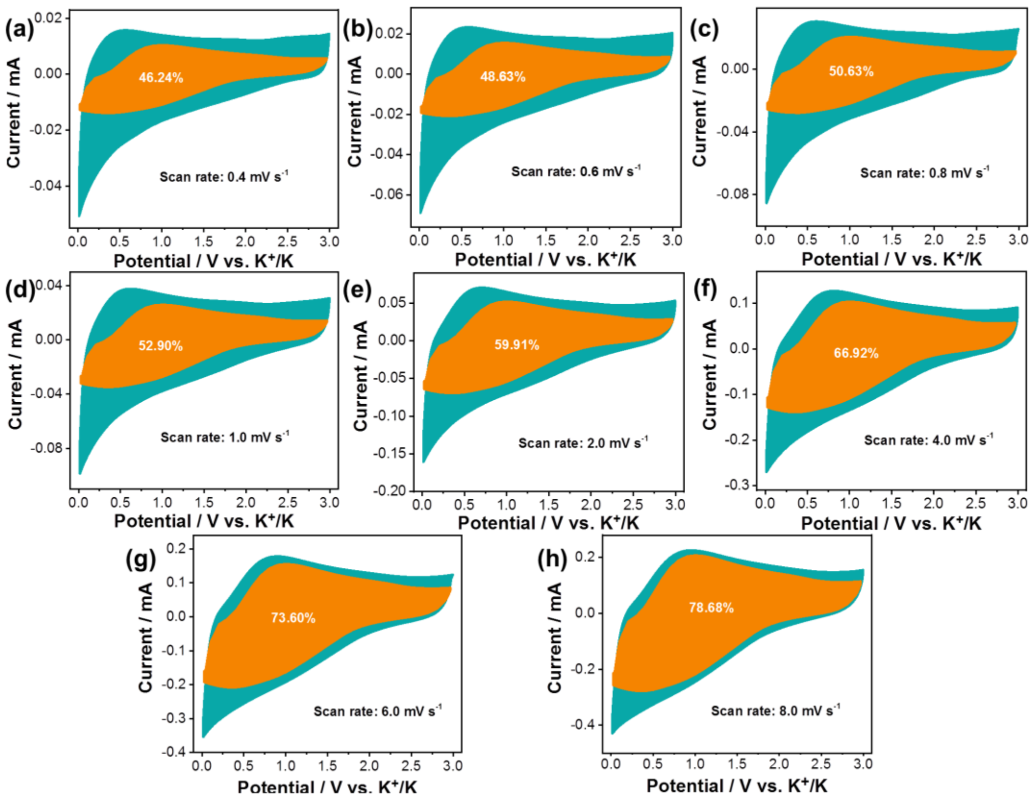

Fig. S18 a-h Capacitive charge-storage contributions under various scan rates

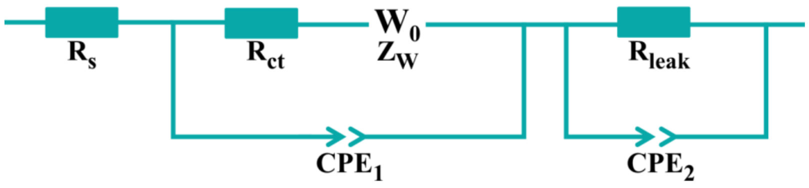

Fig. S19 Equivalent circuit model. R_S presents the equivalent series resistance; Z_w presents the Warburg diffusion element; CPE_1 presents capacitor elements from double layer and active material; R_{leak} is the leakage resistance associated with the electrode reaction in the bulk; CPE_2 is capacitor elements from double layer and active material.

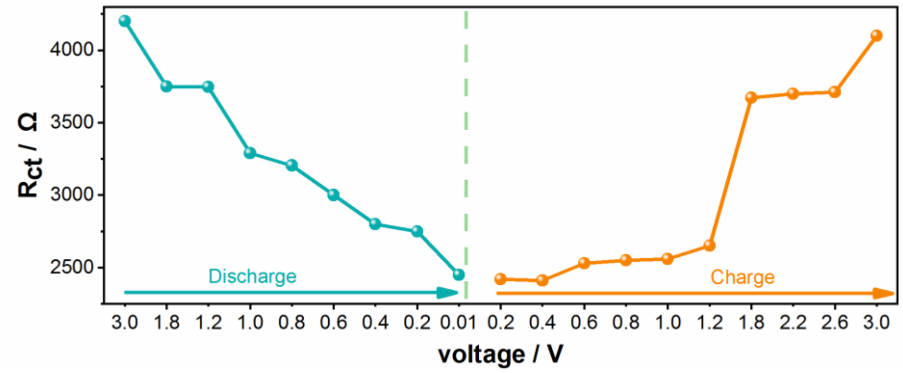

Fig. S20 R_{ct} values based on the ex-situ EIS plots of the discharge process and charge process in the sixth cycle

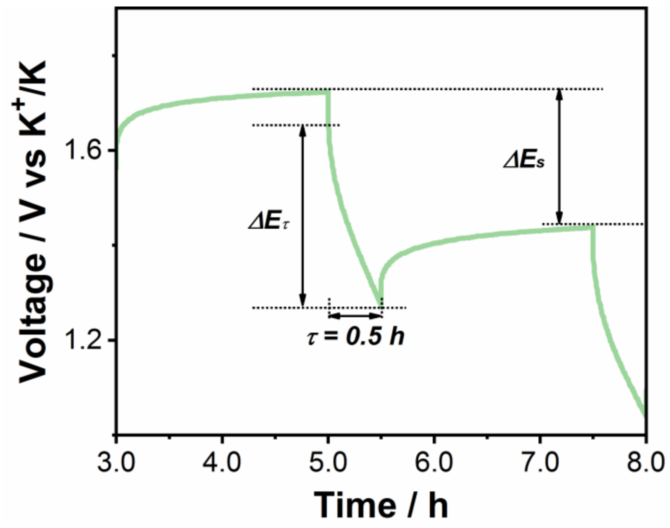

Fig. S21 Schematic diagram of parameter determination based on one step of galvanostatic intermittent titration at 1.72 V vs K^+/K

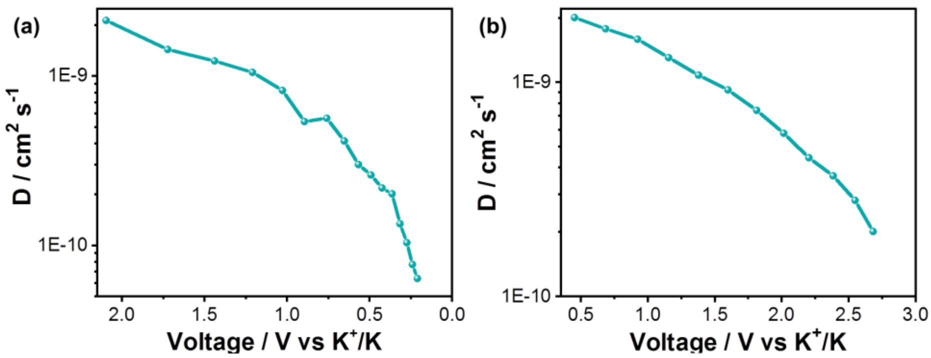

Fig. S22 a Diffusion coefficients of the MCS-7-900 electrode calculated from the GITT curves during discharging process. **b** Diffusion coefficients of the MCS-7-900 electrode calculated from the GITT curves during charging process

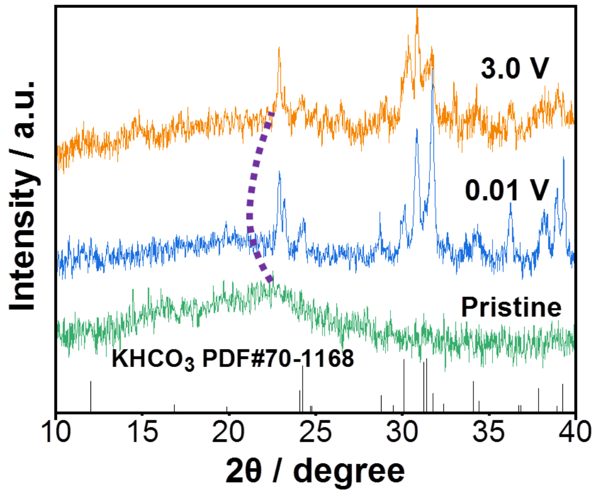

Fig. S23 Ex-situ XRD of three status, namely pristine, discharge to 0.01 V and charge to 3.0 V

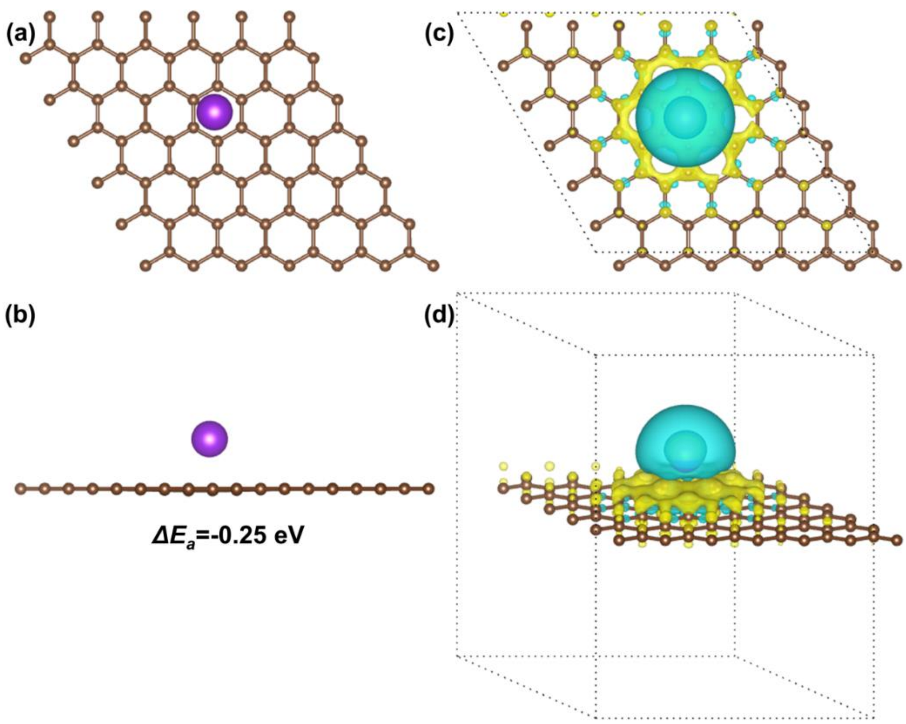

Fig. S24 Theoretical simulations of K-adsorption in the pristine carbon structure. Top and side view of a single K atom adsorbed in the pristine carbon structure (**a, b**). Top and side view of Electron density differences of K absorbed in the pristine carbon structure (**c, d**). Not that, C, N and K atoms are presented by brown, silver and purple balls, respectively. Increased and decreased electron densities are presented by yellow and blue regions, respectively

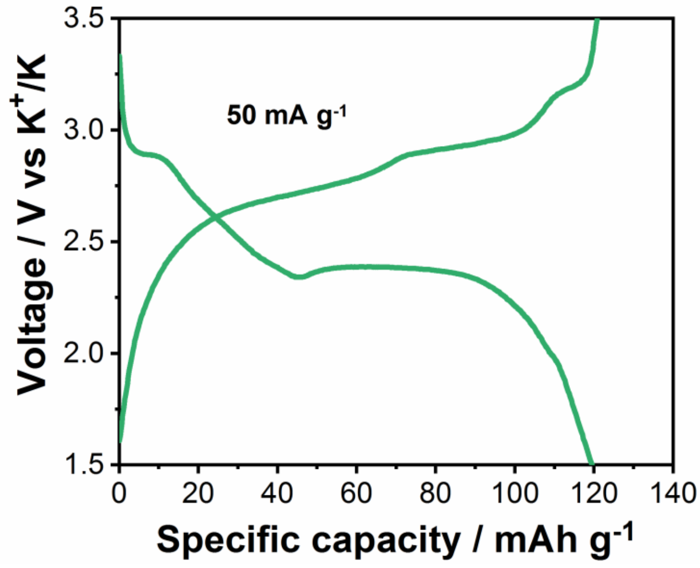

Fig. S25 Charge-discharge profile of PTCDA cathode at 50 mA g^{-1}

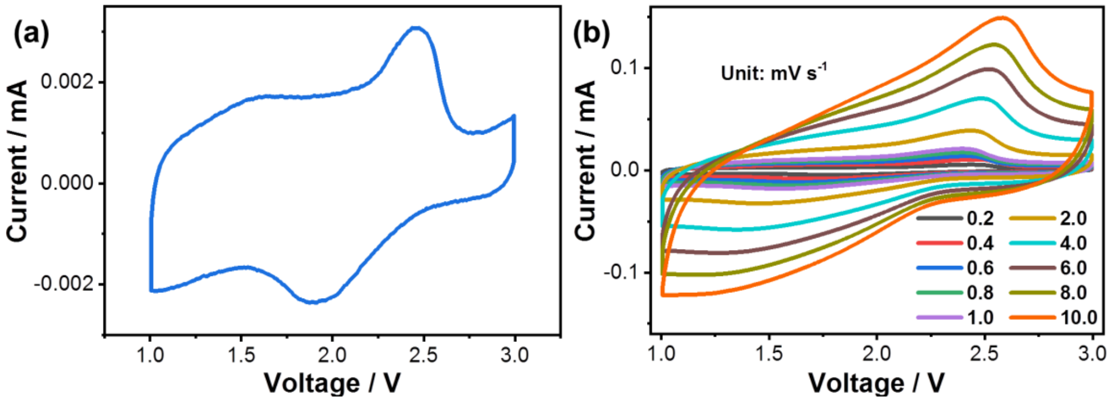

Fig. S26 a CV curve of MCS-7-900//PTCDA at 0.1 mv s^{-1}. **b** The CV curves of MCS-7-900//PTCDA at various current densities.

Table S2 Comparison of the as-obtained full cell and other reported full cells about rate performance and cycling performance

Anode material	Cathode material	Rate performance	Cycling performance	Refs.
MCS-7-900	PTCDA	85.4 mAh g^{-1} at 1000 mA g^{-1}	62.7 mAh g^{-1} after 200 cycles at 1000 mA g^{-1}	This work
Soft carbon	$K_{0.7}Fe_{0.5}Mn_{0.5}O_2$	48 mAh g^{-1} at 100 mA g^{-1}	≈36 mAh g^{-1} after 250 cycles at 100 mA g^{-1}	[S11]
Soft carbon	PI@G	-	≈51 mAh g^{-1} after 80 cycles at 50 mA g^{-1}	[S12]
Soft carbon	P2-$K_{0.44}Ni_{0.22}Mn_{0.78}O_2$	61.3 mAh g^{-1} at 500 mA g^{-1}	≈62 mAh g^{-1} after 200 cycles at 50 mA g^{-1}	[S13]
Hard carbon	s-KCO	36 mAh g^{-1} at 320 mA g^{-1}	≈56.8 mAh g^{-1} after 100 cycles at 30 mA g^{-1}	[S14]
Graphite	$K_{0.6}CoO_2$	-	≈27 mAh g^{-1} after 5 cycles at 3 mA g^{-1}	[S15]
HC/CB	$K_{0.3}MnO_2$	-	46 mAh g^{-1} after 100 cycles at 32 mA g^{-1}	[S16]
Graphite	KNHCF	16.4 mAh g^{-1} at 200 mA g^{-1}	37.8 mAh g^{-1} after 100 cycles at 10 mA g^{-1}	[S17]
Graphite	P3–$K_{0.5}MnO_2$ HSMS	8.2 mAh g^{-1} at 300 mA g^{-1}	40.2 mAh g^{-1} after 50 cycles at 10 mA g^{-1}	[S18]

V_2O_{3-x}@rGO	KVO@rGO	40.7 mAh g^{-1} at 200 mA g^{-1}	38.6 mAh g^{-1} after 250 cycles at 100 mA g^{-1}	[S19]
MoS_2@rGO	$K_2Fe[Fe(CN)_6]$	-	50 mAh g^{-1} after 50 cycles at 50 mA g^{-1}	[S20]
KMO/CNT-30	KMO/CNT-30	-	47.3 mAh g^{-1} after 100 cycles at 100 mA g^{-1}	[S21]

Supplementary References

[S1] X. Zhao, D. Zhou, M. Chen, J. Yang, L.-Z. Fan. Achieving the robust immobilization of CoP nanoparticles in cellulose nanofiber network-derived carbon via chemical bonding for a stable potassium ion storage. RSC Adv. **10**(72), 44611-44623 (2020). https://doi.org/10.1039/D0RA09478A

[S2] M. Wang, Y. Zhu, Y. Zhang, T. Yang, J. Duan et al., Cost-effective hard-soft carbon composite anodes with promising potassium ions storage performance. Electrochim. Acta **368**, 137649 (2021). https://doi.org/10.1016/j.electacta.2020.137649

[S3] X. Shi, Y. Zhang, G. Xu, S. Guo, A. Pan et al., Enlarged interlayer spacing and enhanced capacitive behavior of a carbon anode for superior potassium storage. Sci. Bull. **65**(23), 2014-2021 (2020). https://doi.org/10.1016/j.scib.2020.07.001

[S4] W. Li, Z. Li, C. Zhang, W. Liu, C. Han et al., Hard carbon derived from rice husk as anode material for high performance potassium-ion batteries. Solid State Ionics **351**, 115319 (2020). https://doi.org/10.1016/j.ssi.2020.115319

[S5] X. Shi, Z. Xu, C. Han, R. Shi, X. Wu et al., Highly dispersed cobalt nanoparticles embedded in nitrogen-doped graphitized carbon for fast and durable potassium storage. Nano-Micro Lett. **13**(1), 21 (2020). https://doi.org/10.1007/s40820-020-00534-x

[S6] M. Wang, Y. Zhu, Y. Zhang, J. Duan, K. Wang et al., Isotropic high softening point petroleum pitch-based carbon as anode for high-performance potassium-ion batteries. J. Power Sources **481**, 228902 (2021). https://doi.org/10.1016/j.jpowsour.2020.228902

[S7] H. Wang, A. Artemova, G. Yang, H. Wang, L. Zhang et al., Lotus root-like porous carbon for potassium ion battery with high stability and rate performance. J. Power Sources **466**, 228303 (2020). https://doi.org/10.1016/j.jpowsour.2020.228303

[S8] X. Li, Y. Lei, L. Qin, D. Han, H. Wang et al., Mildly-expanded graphite with adjustable interlayer distance as high-performance anode for potassium-ion

batteries. Carbon **172**, 200-206 (2021). https://doi.org/10.1016/j.carbon.2020.10.023

[S9] K. Cao, S. Wang, Y. Jia, D. Xu, H. Liu et al., Promoting K ion storage property of SnS_2 anode by structure engineering. Chem. Eng. J. **406**, 126902 (2021). https://doi.org/10.1016/j.cej.2020.126902

[S10] M. Yang, L. Jin, M. He, Z. Yi, T. Duan et al., SiOx@C composites obtained by facile synthesis as anodes for lithium- and potassium-ion batteries with excellent electrochemical performance. Appl. Surf. Sci. **542**, 148712 (2021). https://doi.org/10.1016/j.apsusc.2020.148712

[S11] X. Wang, X. Xu, C. Niu, J. Meng, M. Huang et al., Earth abundant Fe/Mn-based layered oxide interconnected nanowires for advanced K-ion full batteries. Nano Lett. **17**(1), 544-550 (2017). https://doi.org/10.1021/acs.nanolett.6b04611

[S12] Y. Hu, H. Ding, Y. Bai, Z. Liu, S. Chen et al., Rational design of a polyimide cathode for a stable and high-rate potassium-ion battery. ACS Appl. Mater. Interfaces **11**(45), 42078-42085 (2019). https://doi.org/10.1021/acsami.9b13118

[S13] X. Zhang, Y. Yang, X. Qu, Z. Wei, G. Sun et al., Layered P2-Type $K_{0.44}Ni_{0.22}Mn_{0.78}O_2$ as a high-performance cathode for potassium-ion batteries. Adv. Funct. Mater. **29**(49), 1905679 (2019). https://doi.org/10.1002/adfm.201905679

[S14] T. Deng, X. Fan, C. Luo, J. Chen, L. Chen et al., Self-templated formation of P2-type $K_{0.6}CoO_2$ microspheres for high reversible potassium-ion batteries. Nano Lett. **18**(2), 1522-1529 (2018). https://doi.org/10.1021/acs.nanolett.7b05324

[S15] H. Kim, J.C. Kim, S.-H. Bo, T. Shi, D.-H. Kwon et al., K-ion batteries based on a P2-Type $K_{0.6}CoO_2$ cathode. Adv. Energy Mater. **7**(17), 1700098 (2017). https://doi.org/10.1002/aenm.201700098

[S16] C. Vaalma, G. A. Giffin, D. Buchholz, S. Passerini. Non-aqueous K-ion battery based on layered $K_{0.3}MnO_2$ and hard carbon/carbon black. J. Electrochem. Soc. **163**(7), A1295-A1299 (2016). https://doi.org/10.1149/2.0921607jes

[S17] S. Chong, Y. Wu, S. Guo, Y. Liu, G. Cao. Potassium nickel hexacyanoferrate as cathode for high voltage and ultralong life potassium-ion batteries. Energy Storage Materials. **22**, 120-127 (2019). https://doi.org/10.1016/j.ensm.2019.07.003

[S18] B. Peng, Y. Li, J. Gao, F. Zhang, J. Li et al., High energy K-ion batteries based on P3-Type $K_{0.5}MnO_2$ hollow submicrosphere cathode. J. Power Sources **437**, 226913 (2019). https://doi.org/10.1016/j.jpowsour.2019.226913

[S19] Z. Tong, R. Yang, S. Wu, D. Shen, T. Jiao et al., Defect-engineered vanadium

trioxide nanofiber bundle@graphene hybrids for high-performance all-vanadate Na-ion and K-ion full batteries. J. Mater. Chem. A. **7**(33), 19581-19588 (2019). https://doi.org/10.1039/C9TA06538E

[S20] S. Chong, L. Sun, C. Shu, S. Guo, Y. Liu et al., Chemical bonding boosts nano-rose-like MoS_2 anchored on reduced graphene oxide for superior potassium-ion storage. Nano Energy **63**, 103868 (2019). https://doi.org/10.1016/j.nanoen.2019.103868

[S21] S. Chong, Y. Wu, C. Liu, Y. Chen, S. Guo et al., Cryptomelane-type MnO_2/carbon nanotube hybrids as bifunctional electrode material for high capacity potassium-ion full batteries. Nano Energy **54**, 106-115 (2018). https://doi.org/10.1016/j.nanoen.2018.09.072
